# 0893. High respiratory rate favors pulmonary edema in an experimental model of acute lung injury

**DOI:** 10.1186/2197-425X-2-S1-O19

**Published:** 2014-09-26

**Authors:** J Retamal, JB Borges, F Suarez-Sipmann, A Bruhn, G Hedenstierna, A Larsson

**Affiliations:** Hedenstierna Laboratory, Uppsala University, Department of Surgical Sciences, Uppsala, Sweden; Pontificia Universidad Católica de Chile, Facultad de Medicina, Departamento de Medicina Intensiva, Santiago, Chile; Hedenstierna Laboratory, Uppsala University, Department of Medical Sciences, Clinical Physiology, Uppsala, Sweden

## Introduction

The ARDS-net protocol [1], recommends that respiratory rate (RR) could be increased at hypercapnia in order to normalize PaCO2. However, **i** n heterogeneously inflated lungs, e.g., ARDS, at every breath the local alveolar distending forces will be amplified up to 4.5 times in the interphase between collapsed and aerated areas [2]. Thus, a higher RR could exaggerate the cyclic deformations of lung parenchyma and might therefore induce further lung injury. Indeed, animal studies using simultaneous modifications of flow and tidal volume have indicated that low respiratory rates are lung protective [3]. We therefore hypothesized that an isolated increase of RR would augment the development of ventilator induced lung injury (VILI).

## Objectives

To compare VILI development at two clinically relevant RR during protective mechanical ventilation setting, keeping constant flow, tidal volume (VT) and pCO2 levels.

## Methods

Healthy piglets were subjected to a two-hit lung injury model (saline lavages followed by 2 hours of injurious ventilation), and then randomized into two groups: LRR 20 breaths/min (n=6), and HRR 40 breaths/min (n=6), and were mechanically ventilated during six hours according to ARDSnet protocol (VT 6 ml/kg, PEEP10 cmH2O, FiO2 0.5), keeping an inspiratory time of 0.5 sec. We used instrumental dead space to keep similar values of pCO2 in both groups. We assessed respiratory mechanics, invasive systemic and pulmonary arterial pressures, volumetric capnography and extravascular lung water (EVLW). At the end of the experiments lungs were excised and wet/dry (W/D) ratio was evaluated.

## Results

**Figure 1:**
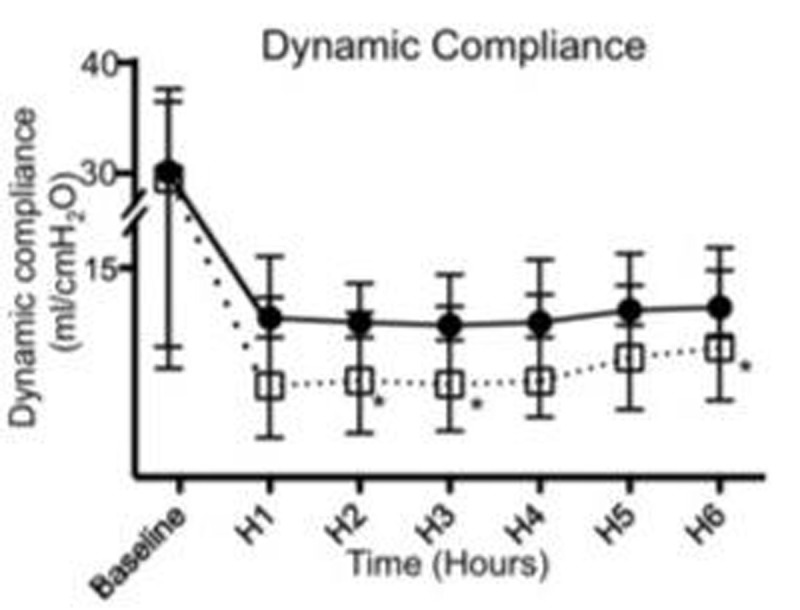
Dynamic compliance

**Figure 2:**
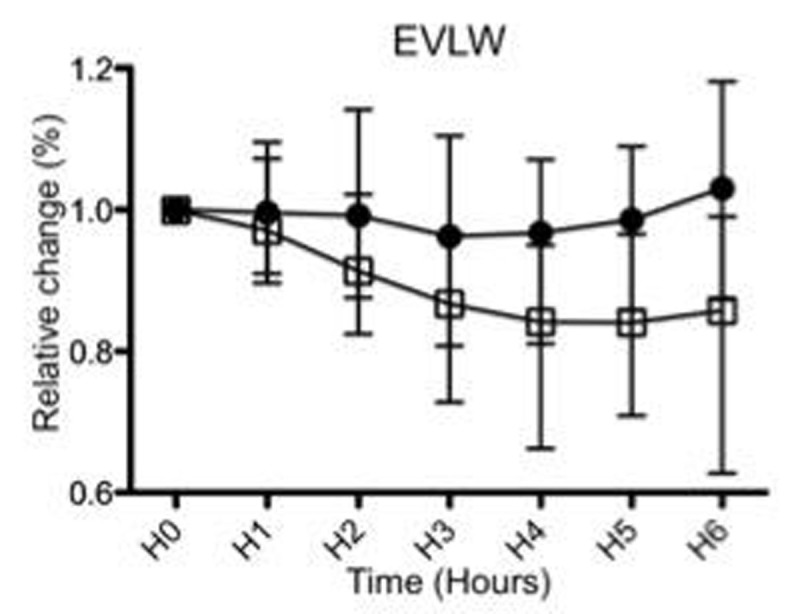
Extravascular lung water (EVLW)

**Figure 3:**
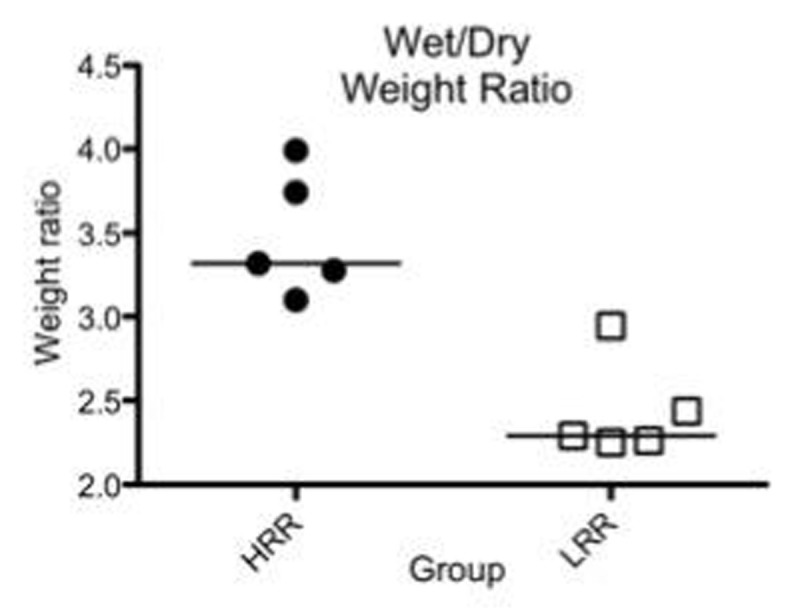
Wet/Dry weight ratio

Baseline data were similar between groups. No differences in oxygenation, pCO2 levels, or in systemic and pulmonary arterial pressures were observed during the protocol. We observed an increase in dynamic compliance (Fig. 1) and a decrease in EVLW (Fig. 2) over time in the LRR group (p< 0.05), but not in the HRR group. In addition, W/D ratio (Fig. 3) was higher in the HRR group (p< 0.05). Data are expressed as median and ranges.

## Conclusions

In our study high respiratory rate reduced lung water clearance, which resulted in an increase of lung water content, indicating that increasing respiratory rate could augment VILI.
